# Treatment landscape of patients with HER2+ early breast cancer: an overview

**DOI:** 10.3332/ecancer.2024.1787

**Published:** 2024-10-10

**Authors:** Frederic Ivan L Ting

**Affiliations:** 1 Department of Clinical Sciences, College of Medicine, University of St. La Salle, Bacolod 6100, Philippines; 2 Department of Internal Medicine, Corazon Locsin Montelibano Memorial Regional Hospital, Bacolod 6100, Philippines

**Keywords:** breast cancer, HER2+, treatment, Philippines

## Abstract

Breast cancer is the most common malignancy in terms of incidence and is the leading cause of cancer deaths among women worldwide. In the Philippines, 33,079 new cases of breast cancer were documented in 2020 comprising 17.5% of all new cancer diagnoses. With a rate of 27 deaths per 100,000 people, the Philippines is the frontrunner in Asia for breast cancer mortality. HER2/neu-positive breast cancer, a more aggressive subtype associated with poorer survival outcomes, is present in about 23.5%. Fortunately, the emergence of HER2-targeted therapies has considerably improved disease-free survival and overall survival. This article reviews the most recent data in the HER2+ early breast cancer space.

## Introduction

Breast cancer is the most common malignancy in terms of incidence and is the leading cause of cancer deaths among women worldwide [1]. In the Philippines, 33,079 new cases of breast cancer were documented in 2020 comprising 17.5% of all new cancer diagnoses [21]. It was reported that 1 out of 13 Filipino women is expected to develop breast cancer in their lifetime [24]. With a rate of 27 deaths per 100,000 people [14], the Philippines is the frontrunner in Asia for breast cancer mortality. HER2/neu-positive breast cancer, a more aggressive subtype associated with poorer survival outcomes [10], is present in about 23.5% [7]. Fortunately, the emergence of HER2-targeted therapies has considerably improved disease-free survival and overall survival [6, 17]. Thus, it is crucial that we review the most recent data in the HER2+ early breast cancer (eBC) space.

## Defining high-risk population

Patients with HER2+ eBC with a higher risk of recurrence should be treated with neoadjuvant therapy plus anti-HER2 targeted treatment. These patients are defined as having tumours of >2 cm and/or node-positive disease [18]. Furthermore, the presence of residual disease following neoadjuvant treatment has been associated with a higher risk of relapse [22] warranting escalation strategies.

## Neoadjuvant setting

The addition of pertuzumab to trastuzumab-based chemotherapy has been noted to significantly increase pathologic complete response (pCR) as shown in the Neosphere and the TRYPHAENA trials. In the Neosphere trial, a 46% pCR rate was noted with docetaxel, pertuzumab and trastuzumab for 12 weeks [8]. Furthermore, the TRYPHAENA trial led to a 66% pCR rate with the docetaxel, carboplatin, trastuzumab and pertuzumab regimen for six cycles [19]. Furthermore, the ADAPT trial also showed a 90.5% pCR rate in patients with HER2-positive and hormone receptor–negative eBC with neoadjuvant trastuzumab-pertuzumab with weekly paclitaxel for 12 weeks [15].

The PEONY phase 3 randomised clinical trial confirms these findings in the Asian population where it demonstrated statistically significant improvement in the total pCR rate [20].

Since the addition of pertuzumab to trastuzumab clearly improves pCR rates in patients with HER2+ eBC with tumours >2 cm and/or node-positive disease, dual anti-HER2 blockade plus chemotherapy is widely considered as the standard of care [2, 3, 5, 9].

## Adjuvant setting

The APHINITY trial showed that the addition of pertuzumab to trastuzumab and adjuvant chemotherapy resulted in a significant improvement in the 3-year invasive disease-free survival (iDFS). It is important to note that the benefit from pertuzumab was only observed in node-positive patients (92% versus 90.2%; hazard ratio (HR) 0.81; *p* = 0.02) and not in node-negative patients [26]. This benefit was maintained in the 8-year analysis with a 23% decrease in iDFS (HR, 0.77; 95% CI, 0.66 to 0.91) and the absolute risk reduction of 2.6% in the intention-to-treat population [11]. The iDFS benefit of the addition of pertuzumab in HER2+ eBC is maintained, with the benefit continuing in the node-positive cohort, regardless of hormone receptor status. Thus, for patients who undergo surgery first, adjuvant chemotherapy plus trastuzumab-pertuzumab should be considered for patients with nodal involvement.

## Adjuvant therapy for patients with residual disease post-neoadjuvant therapy

The randomised phase III KATHERINE trial showed that the replacement of trastuzumab with the antibody-drug conjugate ado-trastuzumab emtansine (T-DM1) in patients who failed to achieve a pCR after neoadjuvant chemotherapy plus anti- HER2 therapy (either single or dual HER2 blockade) demonstrated an 11% absolute improvement in iDFS (3-year iDFS 88% versus 77% in the T-DM1 and trastuzumab groups, respectively) with an HR of 0.50 (95% CI, 0.39 to 0.64; *p* < 0.001). Subgroup analyses showed a consistent benefit, irrespective of hormone receptor status, the extent of residual disease at surgery and prior single or dual HER2-targeted neoadjuvant therapy [25], making adjuvant T-DM1 the standard of care for patients with residual disease following neoadjuvant therapy.

For patients who achieve pCR after neoadjuvant treatment, most guidelines recommend continuing dual anti-HER2 blockade to complete 1 year in high-risk patients at baseline [2, 3, 5]. This is primarily because patients with a pCR can still experience disease relapse with tumour size and nodal status as independent prognostic factors [12].

## Extended anti-HER2 adjuvant therapy

The phase III ExteNET trial looked at extended adjuvant therapy with neratinib, an irreversible, pan-HER TKI, after 1 year of adjuvant trastuzumab. The study showed that neratinib led to an absolute improvement in 5-year iDFS of 2.5% (90.2% versus 87.7%; HR, 0.73; *p* 5 0.0008), but this was mainly restricted to the hormone receptor–positive subgroup [4]. Thus, expert panels recommend consideration of the use of neratinib in patients with HER2+, hormone-receptor positive, node-positive eBC not previously treated with dual blockade while considering the benefits against its toxicities, with diarrhea being a concern among patients [3, 5].

## Future directions in the Philippines

Aside from financial toxicity, it is important to note that one barrier to the efficient delivery of cancer care in the country is the challenges surrounding the actual patient–provider session which include delays in treatment because of limited clinic or treatment slots per day [13, 23]. Fortunately, these delays can be alleviated by the availability of the subcutaneous form of pertuzumab-trastuzumab to which 85% of patients prefer over the intravenous form mainly due to reduced clinic time and comfort during administration [16].

## Conclusion

The long-term outcomes for our patients with HER2+ eBC can be significantly improved by tailoring our treatment plans based on response to neoadjuvant therapy. This paradigm shift reminds us of the importance of using a preoperative systemic approach with dual anti-HER2 blockade therapy for patients with early or locally advanced disease and shifting to adjuvant T-DM1 for patients with residual disease. Furthermore, the subcutaneous form of these anti-HER2 treatments offer a convenient and preferred option for patients.

## Conflicts of interest

The author has no conflicts of interest in writing this paper.
